# Noninvasive oscillometric cardiac output determination in the intensive care unit – comparison with invasive transpulmonary thermodilution

**DOI:** 10.1038/s41598-017-10527-3

**Published:** 2017-08-30

**Authors:** Alexander Reshetnik, Friederike Compton, Anna Schölzel, Markus Tölle, Walter Zidek, Markus van der Giet

**Affiliations:** Department of Nephrology and intensive care medicine, Charité Universitaetsmedizin, Campus Benjamin Franklin, Hindenburgdamm 30, 12203 Berlin, Germany

## Abstract

Assessment of the cardiac output (CO) is usually performed with invasive techniques requiring specialized equipment in the intensive care unit (ICU). With TEL-O-GRAPH (TG), CO can be derived from the oscillometrically obtained brachial pulse wave during the measurement of brachial blood pressure. CO and stroke volume (SV) determinations with TG were compared with transpulmonary thermodilution measurements with the PICCO system (PICCO) in 38 haemodynamically unstable ICU patients with a total of 84 comparison measurements performed. SV (33.3 ± 9.0 ml/m^2^ vs. 44.3 ± 14.4 ml/m^2^, p < 0.001) and CO (2.7 ± 0.5 l/min/m^2^ vs. 3.8 ± 1.2 l/min/m^2^, p < 0.001) were underestimated significantly with TG and oscillometric brachial systolic blood pressure (BP) was significantly lower and diastolic BP significantly higher than invasive femoral artery pressure. A linear correlation was found between CO dimension and CO underestimation with TG. Correct tracking of CO changes with a fluid challenge was possible in 69.5% of measurements. Oscillometric noninvasive CO is possible in the ICU, but accuracy and precision of this new method are lacking. Implementation of a correction factor accounting for the linear increase in CO underestimation observed with increasing CO could improve CO assessment with TG in haemodynamically unstable patients.

## Introduction

Cardiac output (CO) is a major determinant of organ perfusion, and CO monitoring therefore an integral part of the care for haemodynamically compromised patients in the intensive care unit (ICU). Clinical gold standard for CO determination is intermittent thermodilution measurement either performed via a pulmonary artery catheter, or, less invasively, with the PICCO system using the transpulmonary approach with indicator detection in the systemic arterial circulation^1, 2^. Beat-to-beat CO monitoring is possible using CO calculation algorithms derived from the arterial pulse wave, and can either be performed invasively or noninvasively^2, 3^. To date, noninvasive pulse wave recordings have only been established for distal blood pressure measurement sites such as the finger (volume clamp technique) and the radial artery (applanation tonometry)^4, 5^. Distal blood pressure, though, is known to underestimate central arterial pressure in haemodynamically compromised patients, and noninvasive pulse wave derivation is not always possible in patients on vasopressor therapy^6^. With the Tel-O-GRAPH (TG) oscillometric brachial artery blood pressure measuring device, proximal noninvasive arterial pulse wave determination and CO estimation is possible, and the device has been evaluated clinically for the measurement of brachial blood pressure and calculation of central blood pressure and pulse wave velocity in haemodynamically stable patients^7–9^. In the present study, we therefore compared noninvasive CO determination with TG with invasive thermodilution CO measurement in haemodynamically unstable ICU patients to evaluate whether oscillometric TG CO is suitable for use in the ICU.

## Results

A total of 84 TG/PICCO comparison measurements were performed in 38 patients (Table 1). More than one comparison measurement was carried out in 31 patients, and in 33 instances measurements were performed before and immediately after a fluid challenge of 100 ml normal saline intravenously. Demographic data of the patients are displayed in Table 2. At time of study entry, 95 percent of patients were mechanically ventilated, and 76 percent were on continuous vasopressor support because of haemodynamic instability.

**Table 1:** TG/PICCO comparison measurements (n = 84).

	number of patients	number of measurements	number of measurements with fluid challenge^#^
single measurement	7	7	
single measurement on two separate days	5	10	
two measurements before and after fluid challenge on the same day	20	40	20
two measurements before and after fluid challenge on two separate days	4	16	8
two measurements before and after fluid challenge on four separate days	1	8	4
two measurements before and after fluid challenge on day one, single measu-rement on day two	1	3	1
	38	84	33
total			

^#^fluid challenge: 100 ml normal saline intravenously.

**Table 2:** Patient characteristics (n = 38).

age, mean ± SD, years	68.2 ± 12.3
male gender, n (%)	26 (68.4%)
body surface area, mean ± SD, m^2^	1.9 ± 0.2
body mass index mean ± SD, kg/m^2^	26.4 ± 4.8
arm circumference, mean ± SD, cm	29.2 ± 4.3
mortality, n (%)	
at 28-days	18 (47.4%)
at 3 month	24 (63.2%)
SAPS on ICU admission, mean ± SD	57.7 ± 20.6
SOFA at study entry, mean ± SD	11.2 ± 3.9
mechanical ventilation, n (%)	34 (89.5%)
on ICU admission	34 (89.5%)
at study entry	36 (94.7%)
vasopressor therapy at study entry, n (%)	29 (76.4)
acute kidney injury at study entry, n (%)	28 (73.7)
on renal replacement therapy	23 (60.5)
chronic illness, n (%)	
hypertension	17 (44.7)
smoker	7 (18.4)
stroke	10 (26.3)
coronary heart disease	10 (26.3)
myocardial infarction	8 (21.1)
congestive heart failure	7 (18.4)
peripheral vascular disease	5 (13.2)
diabetes mellitus, insulin-dependent	4 (10.5)
diabetes mellitus, non-insulin-dependent	10 (26.3)

SD = standard deviation, ICU = intensive care unit, SAPS = simplified acute physiology score, SOFA = sepsis-related organ failure score.

**Figure 1:**
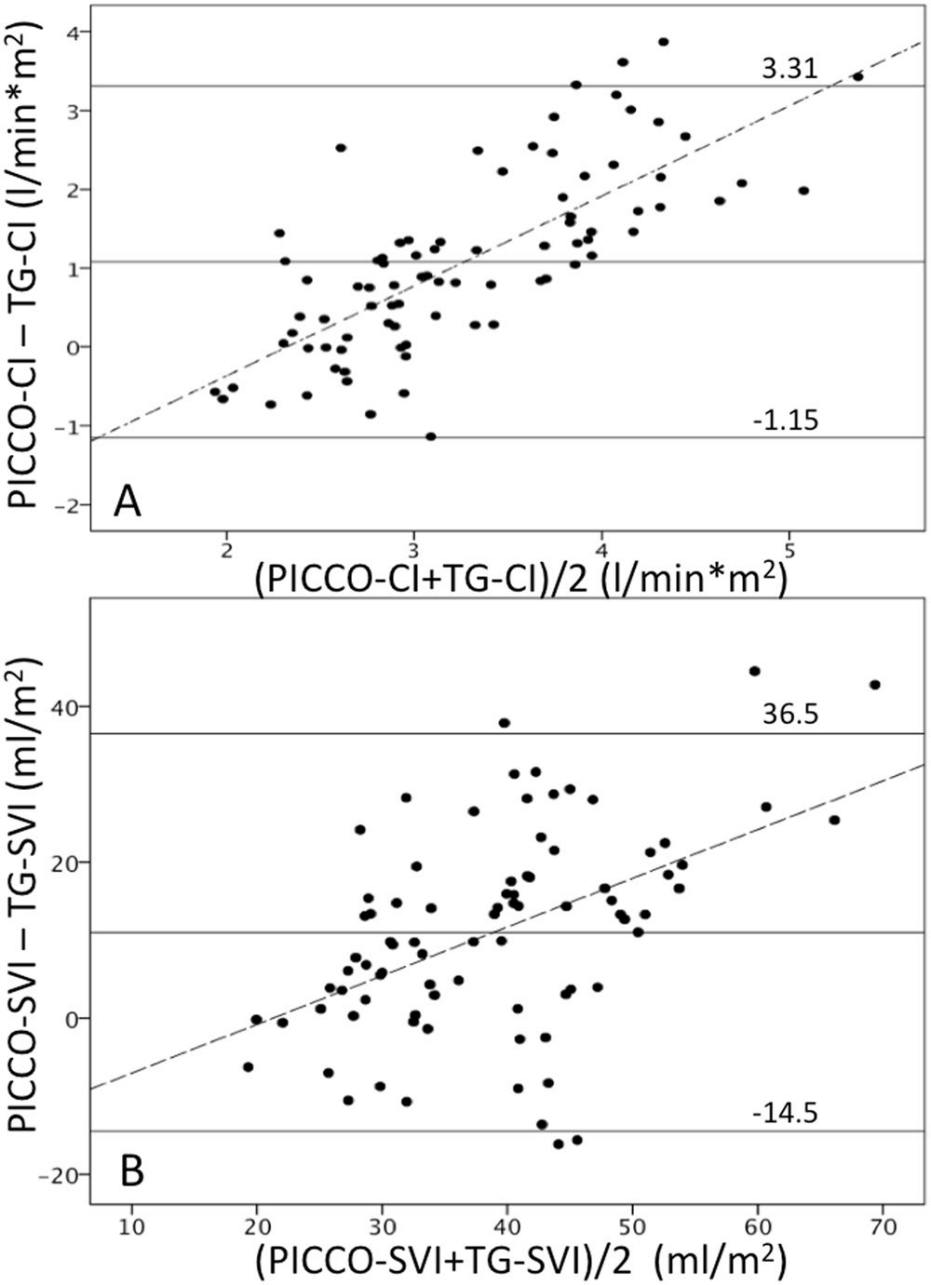
(**A**) Agreement between invasive PICCO measured cardiac index (PICCO-CI) and noninvasive TEL-O-GRAPH derived cardiac index (TG-CI): bias 1.08 l/min/m^2^, limits of agreement −1.15 to 3.31 l/min/m^2^ (percentage error 68.3%), dashed line represents the linear correlation between absolute CI level and PICCO- and TG-CI differences (Pearson’s r = 0.75); (**B**) Agreement between invasive PICCO measured stroke volume index (PICCO-SVI) and noninvasive TEL-O-GRAPH derived stroke volume index (TG-SVI):bias 11 ml/m^2^, limits of agreement −14.5 to 36.5 ml/m^2^ (percentage error 65.7%), dashed line represents the linear correlation between absolute SVI level and PICCO- and TG-SVI differences (Pearson’s r = 0.49).

Mean cardiac index (CI) and stroke volume index (SVI) determined by noninvasive oscillometric TG calculation both were significantly and clinically relevantly lower than PICCO-CI and PICCO SVI measured with transpulmonary thermodilution (TG-CI 2.7 ± 0.5 l/min/m^2^ vs. PICCO-CI 3.8 ± 1.2 l/min/m^2^, p < 0.001 and TG-SVI 33.3 ± 9.0 ml/m^2^ vs. PICCO-SVI 44.3 ± 14.4 ml/m^2^, p = 0.001) (Table 3). Kolmogorov-Smirnov-Test revealed normal distribution for CI- and SVI- between-method bias. Bland-Altman analysis yielded a bias of 1.08 l/min/m^2^ between noninvasive TG- and invasive PICCO-CI measurements, respectively (Fig. 1, panel A). Limits of agreement were ± 1.96 l/min/m^2^ with a percentage error of 68.3%. Differences between TG-CI and PICCO-CI increased in a linear fashion (slope = 1.14) with increasing CI as shown by the dashed line in the Bland-Altman plot (Pearson’s r = 0.75). For SVI, bias was 11 ml/m^2^ with limits of agreement of ±25.5 ml/m^2^ (percentage error 65.7%).

**Table 3:** Comparison of haemodynamic parameters measured noninvasively with TEL-O-GRAPH (TG) and invasively with the PICCO system (PICCO).

parameter	PICCO mean ± SD	TG mean ± SD	p value^#^
heart rate*, /min	86.9 ± 19.9	85.9 ± 19.8	0.72
systolic blood pressure, mmHg	123.6 ± 17.4	117.5 ± 16.0	0.006
diastolic blood pressure, mmHg	61.7 ± 10.7	70.6 ± 9.9	<0.001
cardiac output, l/min	7.34 ± 2.30	5.26 ± 0.93	<0.001
cardiac index, l/min/m^2^	3.80 ± 1.22	2.73 ± 0.50	<0.001
stroke volume, ml	85.2 ± 27.2	63.9 ± 16.3	<0.001
stroke volume index, ml/m^2^	44.3 ± 14.4	33.3 ± 9.0	<0.001

SD = standard deviation ^#^Wilcoxon matched pairs test, *heart rate determined with electrocardiography monitoring and TG, respectively.

**Figure 2:**
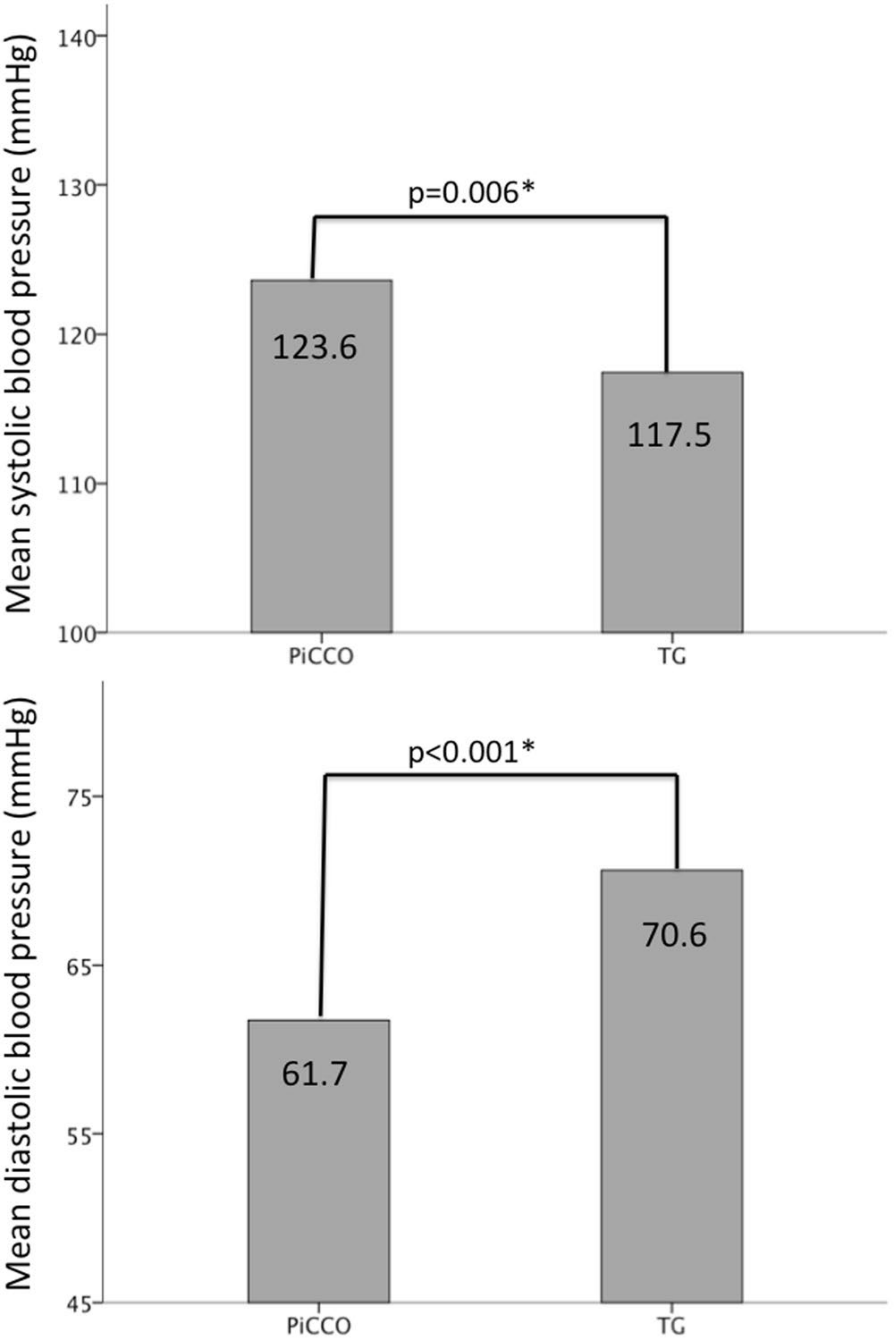
Comparison of mean systolic blood pressure (**A**) and diastolic blood pressure (**B**) measured with the PICCO-system (PICCO) and TEL-O-GRAPH (TG); *-Wilcoxon matched pairs test.

Figure 2 illustrates the mean differences in blood pressure measured noninvasively with TG and invasively with the femoral artery catheter used for PiCCO-CI determination. Systolic blood pressure was significantly lower when measured with TG (117.5 ± 16.0 vs. 123.6 ± 17.4, p = 0.006), whereas diastolic blood pressure was significantly higher with TG than measured intraarterially (70.6 ± 9.9 vs. 61.7 ± 10.7, p < 0.001).

**Figure 3:**
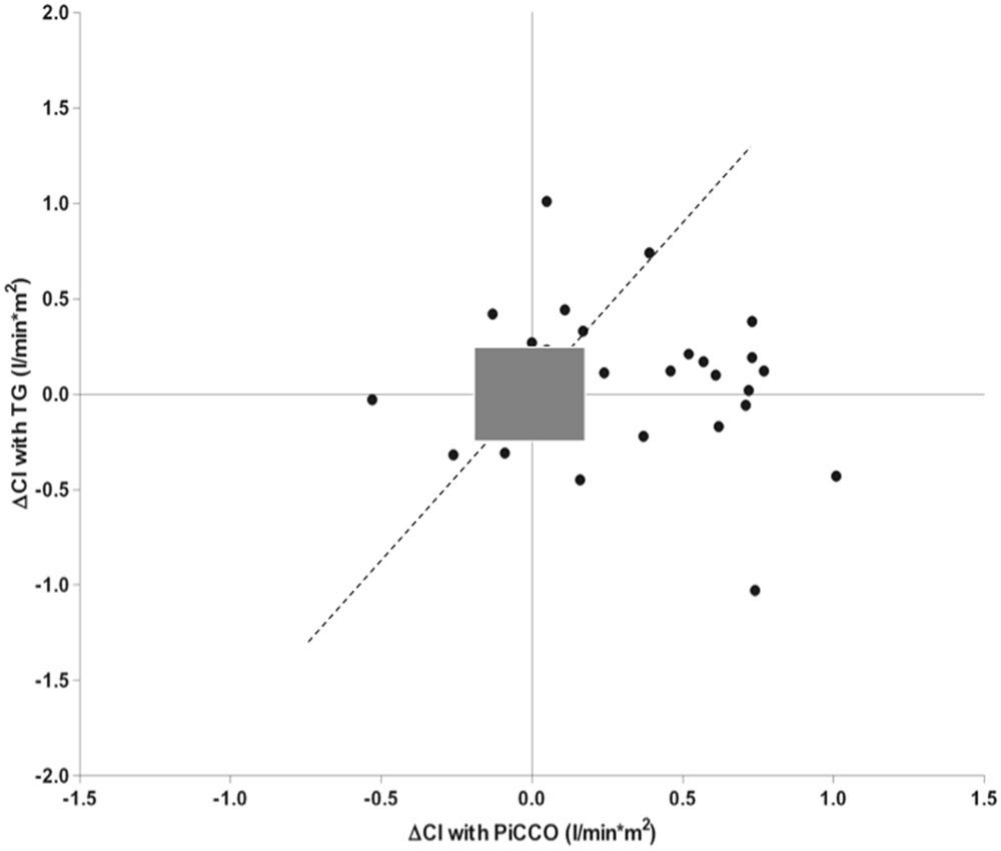
4-quadrant plot of TEL-O-GRAPH (TG) derived cardiac index change (ΔCI) after a fluid challenge of 100 ml normal saline intravenously compared with PICCO measured ΔCI (concordance rate = 69.5%). Points with equal numerical values are located on the 45° diagonal within the quadrant (the dotted line). Exclusion zone is marked by grey rectangle.

When CI changes before and after a fluid challenge of 100 ml normal saline were compared concordant CI increase or decrease was observed in 69.5% of all measurements (Fig. 3). In six instances (18%), there was an increase in TG-CI with fluid challenge, but a decrease or no change with invasive PICCO-CI. In eight measurements (24%), PICCO-CI increased with fluid administration as opposed to a decrease when CI was determined with TG. Comparison of CI changes tracked by TG and reference method is visualized by 4-quadrant plot (Fig. 3).

## Discussion

Our results show that noninvasive oscillometric brachial artery pulse wave acquisition with TG is feasible in haemodynamically unstable ICU patients. TG pulse wave analysis derived SV and CO, though, are clinically relevantly lower when compared to reference thermodilution measurements with the PICCO system.

Different methods of pulse wave analysis can be used to calculate SV from the arterial pressure curve, depending on the underlying model of the circulation and the mathematical transformations performed^10, 11^. Systolic pulse contour analysis as used with TG is based on the Windkessel model, according to which SV is proportional to the area under the systolic portion of the pressure curve^12^. As with all other pulse wave analysis methods, individual aortic impedance has to be taken into account to calculate absolute SV and thus CO values based on this relationship. As opposed to calibrated pulse wave analysis methods, TG uses mathematical transformations only to determine aortic impedance^1, 7^. When compared with calibrated pulse wave analysis, uncalibrated techniques have generally been shown to be less reliable and to underestimate CO especially in haemodynamically unstable patients with constantly changing vascular tone^3, 13–15^. This was recently confirmed in a meta-analysis of uncalibrated noninvasive CO monitoring devices used perioperatively^16^.

With established uncalibrated CO determination devices, the pulse wave is usually obtained from a distal arterial site such as the radial artery or the finger^3^. Blood pressure measurements from peripheral arterial sites have been shown to systematically underestimate central arterial pressure during haemodynamic instability, which in turn influences accuracy and precision of pulse wave derived CO^13, 14, 17^. Reliable blood pressure determination has been shown to be possible with TG in haemodynamically stable ambulatory patients when compared to sphygmomanometric auscultatory blood pressure readings, and oscillometric brachial blood pressure determination is routinely used both in the ICU and the operating theatre even though reliability has not been proven for adult patients in systematic precision and accuracy studies^8, 18–20^. Our data show that despite the more proximal arterial site systolic blood pressure is also underestimated with TG in haemodynamically compromised patients when compared to invasive femoral arterial pressure measurement. Diastolic blood pressure, on the other hand, is overestimated, yielding a deformed pulse wave with diminished pulse pressure used to calculate SV and CO.

To our knowledge, there are only two other studies evaluating the performance of oscillometry-based SV and CO calculation. Liu and coworkers compared oscillometrically derived SV with echocardiographic measurements in 55 haemodynamically stable patients undergoing routine cardiac disease diagnosis^21^. Using a different pulse contour analysis algorithm, the authors found a strong correlation and only minor differences between oscillometry-based and echocardiography-derived SV. Oscillometric SV calculation was evaluated in ten ICU patients in the other study and compared to pulmonary artery thermodilution measurements^7^. Precision and accuracy were reported to be 0.25 ± 12.5 ml (as compared to 21.2 ± 25.1 ml in our study, data not shown), but it was not stated whether TG-SV was higher or lower than thermodilution SV, and only limited data was provided concerning the haemodynamic status of the study subjects. Both studies reported better accuracy of the oscillometric devices that we could show. A possible explanation could be an amount of haemodynamic instability in our patients, which can decrease the sensitivity of peripheral sensors. Thus, final CO/SV calculation can become erroneous.

Bland-Altman analysis of SV and CO data in our study revealed that there was a linear correlation between the dimension of SV and CO, respectively, and the magnitude of underestimation by TG: The higher SV and CO were, the larger the difference between TG and PICCO measurements became. A similar phenomenon had been found in an earlier study performed at our institution comparing applanation tonometry-derived CO and pulmonary artery thermodilution measurements^14^. Because of the linearity of this error it might be possible to develop a correction factor that could be implemented in the TG-CO calculation algorithm allowing for a more precise CO estimation with TG in patients with higher CO levels.

Clinically more important than single absolute CO values often are relative CO changes in response to a therapeutic intervention^22, 23^. CO trending abilities can be assessed using different methods, of which visualization with a 4-quadrant plot and reporting of concordance rates is a method frequently used^24^. We evaluated CO changes with a fluid challenge and found that TG failed to reliably trend CO changes with a concordance rate of 69.5% between TG- and PICCO-ΔCI, which was below an acceptable level of 90–95%^25^. In consistence with the low concordance, 4-quadrant plot visualized low trending ability with a relevant rate of discordant measurements, poor correlation index (r = 0.15) and wide distribution of data points around the 45° diagonal, which represents equal numerical values revealed by TG and PICCO. To our knowledge, there are no other studies assessing the ability of non-invasive oscillometric devices to track CO changes. Several studies addressed this issue for other techniques such as arterial pulse contour cardiac output monitoring systems^11, 26–29^, transtracheal Doppler^30^, thoracic electric bioimpedance monitoring^31^, transesophageal Doppler^25, 32, 33^ with different findings. In general, arterial pulse contour based CO analysis was shown to track CO changes with poor reliability comparable to our results. For other devices, studies showed a better performance. Authors of the published studies used different statistical methods to assess the ability of devices to track CO changes, which makes comparisons between these studies more complicated. Of note, the 4-quadrant plot used in the majority of the published studies as well as in our study, is an excellent tool to visualize the trending ability between the test and reference device. However, it lacks the clearly defined numeric values, which could be used for comparisons between studies, and for the definition of good, acceptable, and poor agreement.

We showed clinically relevant differences in absolute CO values calculated with TG compared to thermodilution measurements. This could be an important factor explaining the poor ability to track CO changes by the tested device. Future studies should be able to show whether improvement in agreement of CO assessment would also improve the ability to track CO changes.

We acknowledge several limitations of our study. Our study group was relatively small, and in most patients more than one measurement was performed and analyzed. Most, but not all patients were on vasopressor support during study measurements. As with all studies comparing two physiologic methods, bias does not necessarily only arise on the part of the method evaluated, but can also be due to inaccuracies of the reference method^34^. Transpulmonary thermodilution CO measurement has been shown to be a reliable method in numerous studies, but the clinical gold standard it was compared with–pulmonary artery thermodilution–likewise has inherent inaccuracies and limitations^35–38^. The character of our study was observational, and apart from the standardized fluid challenge performed when clinically indicated, no intervention was carried out for study purposes. Confounding factors such as positive end-expiratory pressure on mechanical ventilation were therefore not controlled, and the study population was rather heterogeneous. Our study is a method-comparison study of non-invasive CO assessment vs. invasive thermodilution CO measurement as a clinical “gold standard”. Invasive CO monitoring was performed because of the critical illness of our patients. Thus, the findings obtained in our study are limited to this group of critically ill and haemodynamically unstable patients and cannot readily be transferred to other less ill patient collectives.

Despite these limitations, we were able to show that noninvasive oscillometric CO determination is feasible in the ICU. TG does not require specialized equipment as with applanation tonometry or finger blood pressure measurement, and the pulse wave is derived more proximally than with those techniques^39, 40^. With TG, CO can be determined at the same time as the blood pressure is taken, and blood pressure measurement is performed with a method well established in patient monitoring. Future studies will have to show if modifications in the calculation algorithm like the introduction of a correction factor for increasing CO values can improve the performance of oscillometric pulse wave analysis and CO determination.

In conclusion, our study shows that oscillometry-based CO determination is generally possible in haemodynamically unstable ICU patients. Even though precision and accuracy of CO estimation with TG were not sufficient, we were able to demonstrate a linear correlation between the dimension of CO and its underestimation with TG pulse wave analysis. A correction of this systematic error could increase CO determination accuracy significantly, so that oscillometry-derived CO estimation could potentially become an alternative to other more complex noninvasive CO determination methods in the ICU.

## Methods

The study was approved by the Charité Universitätsmedizin Berlin regional research ethics committee (ref: EA1/184/15). All methods were performed in accordance with the relevant guidelines and regulations. Informed consent was obtained from the patient or their legal representative, respectively, before enrollment in the study.

A total of 38 patients treated in the medical ICU of the Charité Campus Benjamin Franklin university hospital in Berlin, Germany between June 2015 and June 2016 and monitored with a PICCO system (Pulsion Medical Systems, Feldkirchen, Germany) as part of their clinical treatment were prospectively enrolled in the study. Exclusion criteria were age below 18 years, pregnancy, known aortic valve, aortic arch, axillary or brachial artery stenosis, as well as cardiac arrhythmias precluding noninvasive calculation of haemodynamic parameters by the TG blood pressure monitoring device. Patients were categorized as haemodynamically unstable if mean arterial pressure (MAP) was <65 mmHg or vasopressor therapy was necessary to maintain MAP ≥ 65 mmHg.

The TG device used in the study was kindly provided by I.E.M., Stolberg, Germany. With TG brachial blood pressure is determined oscillometrically with a conventional brachial blood pressure cuff, and the arterial pulse wave is derived using a high fidelity pressure sensor with the cuff inflated at the diastolic blood pressure level for ten seconds. Estimation of left ventricular stroke volume (SV) and CO is achieved by a series of mathematical transformations of the brachial pulse wave described in detail elsewhere^7^. Briefly, the aortic pressure waveform is calculated using generalized transfer functions (Fourier analysis and de-compensation into wave harmonics), and the aortic flow curve by the means of an adopted, multidimensional Windkessel model. SV is then derived from the time lag between pressure and flow curves, generally referred to as the “characteristic impedance (Zc)”. CO is calculated by multiplying SV with the heart rate also derived from the arterial pulse wave.

With the PICCO system, CO was determined using transpulmonary themodilution. A bolus of 20 mL of cold (0–6 °C) normal saline solution was manually injected (injection time ≤10 seconds) into the distal lumen of a central venous catheter and detected in the systemic circulation by a thermistor-tipped femoral artery catheter (Pulsiocath PV2015L20, Pulsion Medical Systems, Feldkirchen, Germany). CO was calculated as the mean value of three consecutive measurements. To obtain SV, CO was divided by the heart rate determined with electrocardiography (ECG) monitoring.

Noninvasive SV (TG-SV) and CO (TG-CO) were determined at the time of invasive PiCCO CO (PICCO-CO) and SV (PICCO-SV) measurements. For TG measurements, blood pressure cuff size was chosen according to the manufacturer’s specifications (cuff sizes for arm circumferences of 24–34 cm and 32–42 cm, respectively – for arm circumference between 32 and 34 cm the smaller cuff was used). If possible, measurements were performed on both the left and the right arm, and TG-SV and TG-CO calculated as the mean of three (unilateral) or six (bilateral) measurements, respectively.

CO and SV results were indexed to body surface area, and are referred to as cardiac index (CI) and stroke volume index (SVI), respectively. At the time of measurements, blood pressures determined both noninvasively with TG at the brachial artery and invasively with the PICCO system in the external iliac artery were recorded as well as heart rate determined with TG and ICU ECG monitoring, respectively. Patient population characteristics and severity of illness scores (simplified acute physiology score, SAPS, and sepsis-related organ failure assessment score, SOFA) were also registered.

Data were analyzed using Graph-Pad Prism 5 (GraphPad Software, La Jolla, CA, USA) and SPSS Statistics 23.0 (IBM, New York, NY, USA). Results are expressed as mean ± standard deviation. Statistical differences between paired measurements were assessed using nonparametric Wilcoxon testing, and a two-sided p value of <0.05 was considered statistically significant. For agreement between invasive PICCO-CI / PICCO-SVI and noninvasive TG-CI /TG-SVI determinations, Bland-Altman analysis was performed calculating bias as the mean difference between paired measurements, and the 95% confidence interval as limits of agreement. Percentage error was then calculated as suggested by Critchley and Critchley^34^. Linear regression analysis was used to evaluate the progressive deviation between PICCO-CI and TG-CI observed with rising CI levels. To compare CI-changes (ΔCI) induced by a fluid challenge and registered with PICCO and TG, respectively, a 4-quadrant plot was generated and the concordance rate determined^41^. For CO, the recommended margin for the exclusion zone in a 4-quadrant plot is 0.5 l/min^24^. To compare CI changes, the exclusion zone was defined by dividing 0.5 l/min by mean body surface area.
